# Oxidative Stress Responses and Gene Transcription of Mice under Chronic-Exposure to 2,6-Dichlorobenzoquinone

**DOI:** 10.3390/ijerph192113801

**Published:** 2022-10-24

**Authors:** Wenjing Wu, Yingying Liu, Chunze Li, Fangyu Zhuo, Zexiong Xu, Huachang Hong, Hongjie Sun, Xianfeng Huang, Xinwei Yu

**Affiliations:** 1 College of Geography and Environmental Science, Zhejiang Normal University, Jinhua 321004, China; 2 National and Local Joint Engineering Research Center for Ecological Treatment Technology of Urban Water Pollution, College of Life and Environmental Science, Wenzhou University, Wenzhou 325035, China; 3 Key Laboratory of Health Risk Factors for Seafood of Zhejiang Province, Zhoushan Municipal Center for Disease Control and Prevention, Zhoushan 316021, China; 4 College of Marine Science and Technology, Zhejiang Ocean University, Zhoushan 316021, China

**Keywords:** 2,6-dichlorobenzoquinone, mouse, oxidative stress, lipid peroxidation, Nrf2-keap1, mRNA transcription

## Abstract

2,6-Dichlorobenzoquinone (2,6-DCBQ), as an emerging disinfection by-production, was frequently detected and identified in the drinking water; however, limited information is available for the toxic effect of 2,6-DCBQ on mice. In the present study, adult mice were used to assess the impact of 2,6-DCBQ via measuring the responses of antioxidant enzymes (superoxide dismutase (SOD) and catalase (CAT)), the key genes (Heme oxygenase-1 (HO-1), NADPH quinone oxidoreductase 1 (NQO1) and glutamate-L-cysteine ligase catalytic subunit (GCLC)) in the Nrf2-keap1 pathway, and lipid peroxidation (malonaldehyde, MDA). Our results clearly indicated that 2,6-DCBQ decreased the activities of SOD and CAT, repressed the transcriptional levels of key genes in Nrf2-keap1 pathway, further caused oxidative damage on mice. These results provided evidence for assessing the threat of 2,6-DCBQ on human.

## 1. Introduction

Disinfection byproducts (DBPs) are a kind of compounds unintentionally produced from the reactions of natural organic matter (NOM) with disinfectants during the water disinfection process [1,2,3]. Hitherto over 700 DBPs have been detected in the drinking water, of them, THM (trihalomethane) and HAA (haloacetic acid) were strictly regulated due to higher health risk on the public [4,5]. Halobenzoquinones (HBQs), as an emerging DBPs, have attracted more attentions due to their higher toxicity potency than THM and HAA. Bull et al. (2007) had predicted that the toxic effect of HBQs is 1000 times higher than that of regulated DBPs (THM and HAA) via quantitative structural toxicity relationship modeling [6]. Thereafter, the higher toxic effects of HBQs were confirmed in many studies. For example, Dr. Li groups manifested that HBQs exert higher cytotoxicity, genotoxicity and developmental toxicity than that of regulated DBPs [7,8,9]. Therefore, the presence of HBQs is considered as a potential threat to public health.

Up to now, 12 kinds of HBQs were identified in the drinking waters, of them, 2,6-DCBQ was proved to be the most toxic and highest levels DBPs [10,11], and many documents recorded the toxic effect of 2,6-DCBQ, for example, Fu et al. (2017) had reported that 2,6-DCBQ can induce cell cycle arrest of human neural stem cells [12], and Chen et al. (2015) had pointed out that 2,6-DCBQ can induce DNA damage in *E. coli* cells [13], our previous studies also manifested that 2,6-BDCQ can cause oxidative damage on zebrafish [14]. Most of these studies considered that 2,6-DCBQ-induced reactive oxygen species (ROS) is a primary contributor to various toxicity. Thus, understanding the performance of oxidative stress caused by 2,6-DCBQ in mouse is important to elucidate the toxic effect of 2,6-DCBQ on human.

Under normal circumstances, there is a balance between oxidative stress and antioxidant system in organisms. While xenobiotic induce organism generate more reactive oxygen species (ROS), meanwhile, the organism correspondingly improved antioxidant capacity to counteract the ROS [15,16]. Superoxide dismutase (SOD) and catalase (CAT) are the first line of antioxidant enzymes, which play a crucial role in the resistance of oxidative stress and are considered as the ideal indicators to indicate the oxidative stress in organism [17,18]. Furthermore, other antioxidant components are also involved in the eliminating ROS, Nrf2, as a redox-sensitive transcription factor in the organism, is a crucial functional molecule involved in ROS scavenging process [19]. In the normal cases, Nrf2 is controlled at very low levels by the negative regulation of keap1, but it can activate a series of downstream antioxidant genes to against oxidative stress. Of downstream genes, Heme oxygenase-1 (HO-1), NADPH quinone oxidoreductase 1 (NQO1) and glutamate-L-cysteine ligase catalytic subunit (GCLC) have been considered as the usual indicators to indicate antioxidant response of organism due to higher capacity in eliminating oxidative stress [20]. When excessive ROS exceeds the antioxidant capacity of the organism, a series of detriment effects will occur, of them, lipid peroxidation is the direct outcome. MDA (malondialdehyde), as the final product of tissue lipid peroxidation, was considered as the typical indicator of oxidative damage [21].

Although previous studies provided some evidence regarding as the toxic effects of 2,6-DCBQ, those results aren’t suitable for human due to higher species difference. Due to sharing similar morphologic structure and the cellular composition with human, mouse is considered as a reliable research model for toxicology research [22,23]. Given different age of mouse represents different age group of humans, the characteristics of adult mouse are analogous to immunologically experienced adult humans. 2,6-DCBQ is an emerging disinfection byproduct, which possesses higher toxic; furthermore, adult human expose to more 2,6-DCBQ than child and elderly, therefore, adult mouse was used to conduct experiment. Our objectives were to: (1) determine the oxidative stress caused by 2,6-DCBQ; (2) determine the oxidative damage on the mice caused by 2,6-DCBQ; (3) determine the impact of 2,6-DCBQ on the transcriptional levels of key genes in Nrf2-keap1 pathway. The result from this study will reveal the toxic effect of 2,6-DCBQ and providing new insight on the toxicity of 2,6-DCBQ.

## 2. Materials and Methods

### 2.1. Chemical

2,6-DCBQ (CH_3_(CH_2_)_3_COCH(CH_3_)_2_, CAS: 13019-20-0, purity ≥ 98%) was purchased from ThermoFisher Scientific Co., Ltd. (Waltham, MA, USA). Ethanol solution (Sigma-Aldrich, Saint Louis, MO, USA) was used to make 10 g L^−1^ stock solution and frozen at −80 °C before use. Different concentrations of test solutions were prepared by dissolving 2,6-DCBQ stock solution into 0.9% saline solution. The reagent kits used in biochemical, and assays were purchased from Nanjing Jiancheng Bioengineering Research Institute (Nanjing, China). The primers, reagents and kits for gene expression measurement were purchased from Sangon Biotech Co., Ltd. (Shanghai, China), and TaKaRa Co., Ltd. (Dalian, China).

### 2.2. Mouse Acclimation and Exposure

The male mouse is more irritable and fights more during adolescence period; therefore, female mice were employed in our study. After obtaining them from Jinhua Experimental Animal Center (Jinhua, China), fifty ICR female mice (4 weeks old, 17 ± 0.55 g body weight) were acclimated to the standard animal house. All mice were cared following the method reported in our previous studies [24]. Briefly, they were housed in plastic cages with dry woodchips for 7 days prior to experiment, with room conditions being kept at 25 ± 1 °C, 60 ± 10% relative humidity, and a 12 h light-dark cycle. During acclimation, the mice were fed with standard chow diet and Milli-Q water ad libitum. All animal care and experimental procedures were in accordance with the principles and guidelines of Zhejiang Normal University, governing the protection of animals.

Different concentrations of 2,6-DCBQ were prepared according to Shi et al. study [25], they have explored the impacts of halogenated benzene at 100, 500, 2000 and 5000 mg/kg, considering halogenated benzoquinone exert higher toxicity potency, so different concentrations (0, 10, 20, 50 and 100 mg/kg) were chose in this study. After acclimation, 50 mice were weight and randomly divided into 5 groups: a control group and 4 treatment groups. These mice were exposed to different concentrations (0, 10, 20, 50 and 100 mg/kg) of 2,6-DCBQ via daily gavage, after fasting for the whole night. During the exposure period, mice in each group continued to receive food and water with free access. After exposure for 30 days, the mice were sacrificed by carbon dioxide anesthesia. The liver was collected and rinsed with physiological saline (0.9%, *w*/*v*), and then stored in a −80 °C refrigerator for following biochemical assays.

### 2.3. Oxidative Stress Analysis

The impacts of 2,6-DCBQ on oxidative stress and damage were determined following the method described in Zhang, et al. [26]. Briefly, ~0.1 g liver tissues of each mouse were taken and homogenized with 0.9 mL physiological saline solution, and three biological replicated experiments. In order to remove cellular debris and cartilage fragments, the homogenates were centrifuged at 4000 rpm for 10 min at 4 °C; the supernatants were carefully collected to serve as the experimental solution to determine the activities of CAT and SOD, and MDA levels. In order to decrease the disruption of external factor, the samples were always kept on ice during the entire procedure. CAT (U mg^−1^ protein), SOD (U mg^−1^ protein), and MDA (U mg^−1^ protein) were determined using the Diagnostic Reagent Kit (Nanjing Jiancheng Bioengineering Institute; Nanjing, China). Furthermore, the protein level of each sample was also measured using the Kit (Coomassie protein assay dye) to quantitate the activities of CAT and SOD, and MDA levels.

### 2.4. RNA Extraction, cDNA Synthesis for Genes and Quantification of Gene Expression

To understand the response of mouse to 2,6-DCBQ-induced ROS, several key genes (Nrf2, Keap1, HO-1, NQ01, GCLC) involved in Nrf2-keap1 pathway were evaluated at molecular levels. Total RNA extraction from livers of mice from each group (*n* = 3) was conducted using Trizol according to the method described in our previous studies [14]. Subsequently, these RNA samples were used to synthesized complementary DNA by PrimeScript RT reagent kit (TaKaRa, Osaka, Japan) following the manufacturer’s instructions (TaKaRa, Osaka, Japan). Real-time quantitative polymerase chain reaction (qPCR) was carried out with LightCycler96 (Roche, Basel, Switzerland). The reaction mixtures included 12.5 μL of SYBR Premix Ex TaqII (TaKaRa), 0.5 μL cDNA, 10 pmole of each forward and reverse primer (Table 1), and 11 μL ddH_2_O. The thermal cycling program was set as follows: initial denaturing at 95 °C for 30 s, followed by 40 cycles of 95 °C for 5 s, and 60 °C for 30 s. Three biological replicated experiments. Three biological replicated experiments, and the gene expressions of these key genes were calculated and normalized against the housekeeping gene β-actin using the equation fold change = 2^−^^∆∆T^ [27].

### 2.5. Statistical Analysis

All statistical analyses were performed using SPSS 21 software (IBM, Armonk, NY, USA). Also, error bars were revealed by standard deviation (mean ± SD). All data are presented as mean ± SD. The data on the activities of CAT and SOD and the contents of MDA were evaluated by one-way analysis of variance followed by Duncan’s multiple range test (a = 0.05). All figures and statistical analyses were carried out with Sigmaplot 11.0.

## 3. Results and Discussion

Although 30 days exposure of 2,6-DCBQ had no significant effect on the mice growth (Table 2), the presence of 2,6-DCBQ in drinking water posed a threat to the mice. In order to understand the impact of 2,6-DCBQ on mice, we further assessed the response of antioxidant system.

### 3.1. The Impact of 2,6-DCBQ on the Activities of SOD and CAT

**Figure 1:**
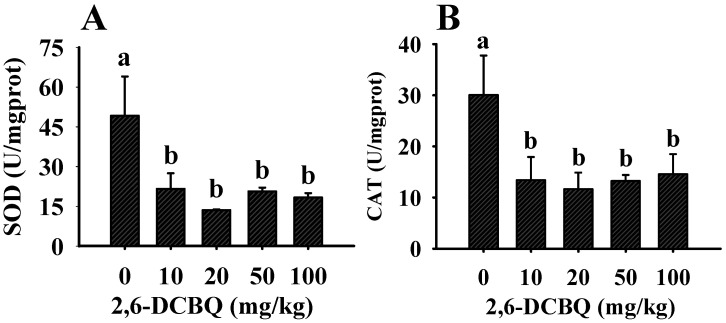
Changes in the activities of SOD (**A**) and CAT (**B**) of mice under different 2,6-DCBQ concentrations after 30 d of exposure. Values are plotted as the mean ± SD, and different letters denote significant difference at *p* < 0.05.

There is a dynamic balance between ROS and antioxidant system in organism, whereas 2,6-DCBQ exposure induced more ROS generation and disrupted the balances. Meanwhile, the organism improved antioxidant capacity to deal with excessive ROS. SOD and CAT, as the high-efficiency antioxidant enzymes, constitute the major defensive system against oxidative stress [28]. As shown in Figure 1, the activities of SOD and CAT displayed a significant decrease in 2,6-DCBQ treatments, compared with the control group. Similar results were reported in Huang, et al. [29], they also found that carbon tetrachloride significantly reduced the activities of SOD and CAT. We speculated that this may be due to the inabilities of SOD and CAT to overcome extremely high levels of ROS, and excessive ROS in turn inactivate SOD and CAT activities.

Collectively, 2,6-DCBQ exposure caused excessive ROS generation. ROS is active, which pose a potential threat to human health; therefore, it is urgent to further evaluate the effect of ROS caused by 2,6-DCBQ.

### 3.2. The Impact of 2,6-DCBQ on the Transcriptional Levels of Nrf2 and Keap1

**Figure 2:**
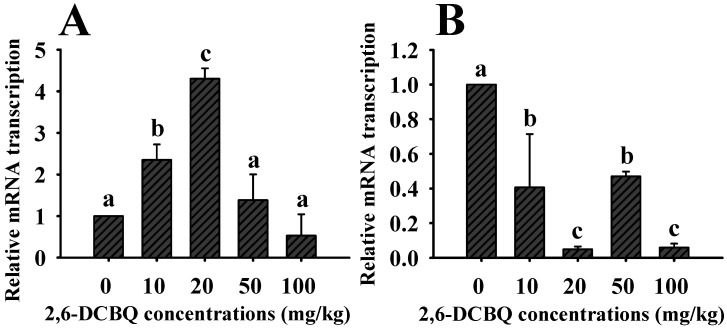
The effects of different concentrations of 2,6-DCBQ on the expressions of Nrf2 (**A**) and keap1 (**B**) of mice, after 30 d of exposure. Values are plotted as the mean ± SD, and different letters denote significant difference at *p* < 0.05.

Nrf2-Keap1 pathway can counteract ROS by controlling some antioxidant genes, which play a crucial role in protecting cells from oxidative stress. Once oxidative stress occurred, Nrf2 is activated from keap1 [30]. In the present study, the transcriptional levels of Nrf2 were significantly elevated at 10 mg/kg and 20 mg/kg 2,6-DCBQ treatments, and it recovered to normal level at 50 mg/kg and 100 mg/kg 2,6-DCBQ treatments (Figure 2). This result indicated that Nrf2 was a positive response to the oxidative stress. It is worth noting that higher concentrations of 2,6-DCBQ caused more severe effect on mice, and Nrf2 transcriptional levels decreased to the control group level with 2,6-DCBQ concentration increasing, probably this was as the result of the excessive high concentration of 2,6-DCBQ had caused severe damage on the organism. Similar phenomenon was also reported in previous studies, Tang, et al. [31] had reported that disc degeneration severe damage reduced the Nrf2 mRNA transcriptional levels. Unlike Nrf2, keap1 transcriptional level was obviously inhibited in this study, this is because keap1, as a regulator of Nrf2, exerts little contribution in antioxidant defense.

In short, 2,6-DCBQ exposure not only decreased the activities of SOD and CAT but also caused the reduction to Nrf2 and Keap1 mRNA transcriptional levels. In order to alleviate oxidative stress, more antioxidant elements were involved in antioxidant reaction, we will further explore the response of key genes in Nrf2-keap1 pathway to understand the impact of 2,6-DCBQ on mice.

### 3.3. The Impact of 2,6-DCBQ on the Transcriptional Levels of HO-1, NQO1 and GCLC

**Figure 3:**
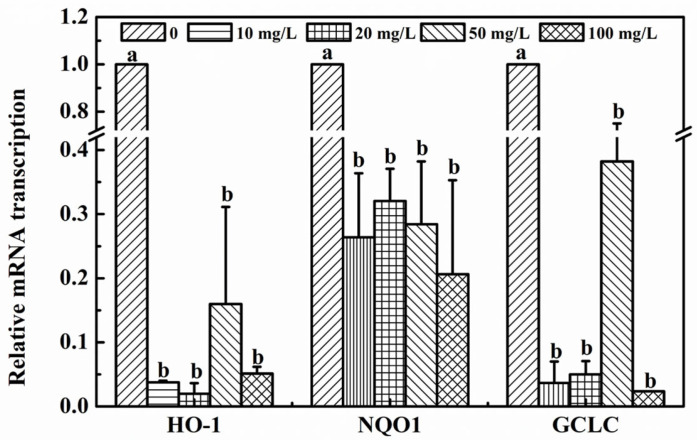
The effect of different concentrations of 2,6-DCBQ on the gene expression of HO-1, NQO1 and GCLC of mice, after 30 d of exposure. Values are plotted as the mean ± SD, and the lowercase letters above the bars represent a significant difference between groups.

Heme oxygenase-1 (HO-1), NADPH quinone oxidoreductase 1 (NQO1) and glutamate-L-cysteine ligase catalytic subunit (GCLC), as the downstream genes in Nrf2-Keap1 pathway, play a crucial role in the cellular defense against oxidative stress in organism [20,30,32]. Of them, HO-1, is a stress response enzyme, which can eliminate oxidative stress via providing biliverdin [33]. In the present study, we found that 2,6-DCBQ had a significant effect on the mRNA transcriptional levels of HO-1 (Figure 3). This result indicated that lots of oxidative stress caused by 2,6-DCBQ exceeded the capacity of antioxidant system and inhibited the HO-1 mRNA transcriptional level. Similar case was reported by Robaczewska, et al. [34], who found that HO-1 mRNA transcriptional level was significantly repressed by high levels of oxidative stress.

For NQO1, it is an endogenous molecule with a conjugated double bond system, which is responsible for maintaining the redox homeostasis in organism [35]. In the present study, after exposure to 2,6-DBCQ, NQO1 mRNA transcriptional level showed a significant decrease of, suggesting that 2,6-DCBQ exposure significantly reduced NQO1 mRNA transcriptional level (Figure 3). We speculated that this probably due to the 2,6-DCBQ cytotoxicity attributed the decrease of NQO1 mRNA transcription, and Butsri et al. [36] had pointed out that NQO1 play a crucial role in maintaining the ability of cell proliferation and replication, and pollutants induced cytotoxicity via inhibiting NQO1 mRNA transcription.

For GCLC, it is the rate-limiting enzyme for GSH synthesis, and GCLC has been considered as the indicator to indirectly reflect the degree of oxidative damage. In this experiment, GCLC mRNA transcription displayed similar variation to that of HO-1 and NQO1 (Figure 3). This probably because that oxidative stress caused by 2,6-DCBQ depleted the GSH content.

In short, HO-1, NQO1 and GCLC mRNA transcriptional levels were inhibited by 2,6-DCBQ. These results indicated that oxidative stress caused by 2,6-DCBQ far exceeded the capacity of antioxidant defense, and probably further induce oxidative damage on the mice. In order to understand the impact of 2,6-DCBQ, further assessment was conducted.

### 3.4. The Impact of 2,6-DCBQ on MDA Levels

**Figure 4:**
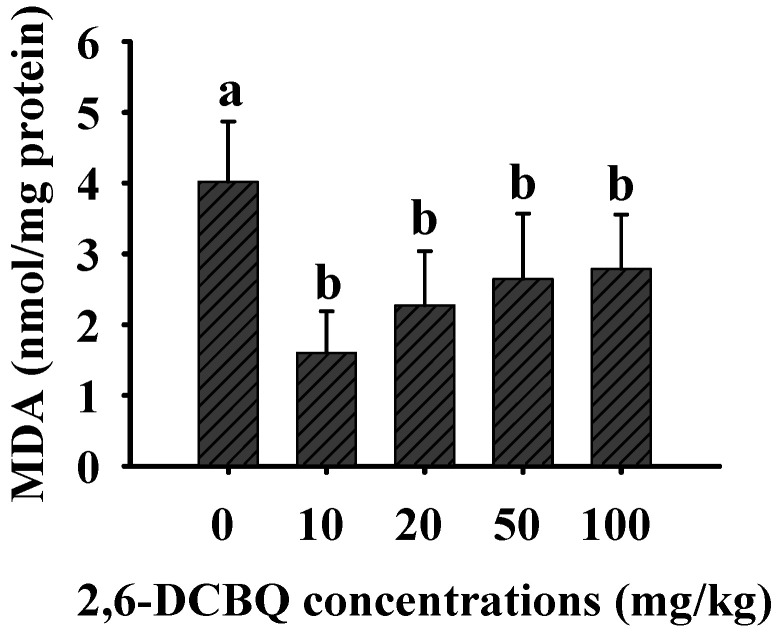
The effects of different concentrations of 2,6-DCBQ on the MDA contents of mice, after 30 d of exposure. Values are plotted as the mean ± SD, and the lowercase letters above the bars represent a significant difference between groups.

Excessive ROS can cause a series of detriment effects on organisms, of these, MDA is considered the most direct outcome of oxidative damage [37]. In the present study, MDA contents in the treatment groups was significantly lower than that in the control group (Figure 4). We speculated that the decrease of MDA level probably because that ≥10 mg kg^−1^ 2,6-DCBQ exerted higher toxic effect, decreasing the total polyunsaturated fatty acids which was prone to peroxidative damage. Similar case was also shown in our previous study, we found that higher concentrations of microcystin-LR decreased MDA levels [28]. This result suggested that higher concentrations of 2,6-DCBQ exert higher toxic effect on mice.

Taken together, 2,6-DCBQ exposure induced severe oxidative damage, and ≥10 mg/kg oral exposure posed a considerable threat to mice.

## 4. Conclusions

Our results clearly indicated that, after exposure to 30 days, 2,6-DCBQ significantly decreased the activities of SOD and CAT, inhibited the transcriptional levels of key genes (HO-1, NQO1 and GCLC) in Nrf2-keap1 pathway. Besides, 2,6-DCBQ also caused oxidative damage to the mice. This study provides novel insight into 2,6-DCBQ-induced toxicity, more work is needed to elucidate the mechanisms for 2,6-BDCQ-induced toxicity.

## Figures and Tables

**Table 1:** Primer used for qPCR in this experiment.

Gene	Type	Sequence
Nrf2	Forward sequence (5′-3′)	5′-CAGTGCTCCTATGCGTGAA-3′
	Reverse sequence (5′-3′)	5′-GCGGCTTGAATGTTTGTC-3′
Keap1	Forward sequence (5′-3′)	5′-CTGGTATCTGAAACCCGTCTA-3′
	Reverse sequence (5′-3′)	5′-TGGCTTCTAATGCCCTGA-3′
HO-1	Forward sequence (5′-3′)	5′-ACAGATGGCGTCACTTCG-3′
	Reverse sequence (5′-3′)	5′-TGAGGACCCACTGGAGGA-3′
NQ01	Forward sequence (5′-3′)	5′-CTTTAGGGTCGTCTTGGC-3′
	Reverse sequence (5′-3′)	5′-CAATCAGGGCTCTTCTCG-3′
GCLC	Forward sequence (5′-3′)	5′-GGATGATGCCAACGAGTC-3′
	Reverse sequence (5′-3′)	5′-GTGAGCAGTACCACGAATA-3′
β-actin	Forward sequence (5′-3′)	5′-CTGTCCCTGTATGCCTCTG-3′
	Reverse sequence (5′-3′)	5′-TGGCTTCTAATGCCCTGA-3′

**Table 2:** The effects of different concentrations of 2,6-DCBQ on weight gain of mice after 30 d of exposure.

Treatment (mg kg^−1^)	0	10	20	50	100
Weight gain (g)	17.4 ± 5.12	17.6 ± 2.51	17.7 ± 3.25	15.4 ± 2.0	16.8 ± 2.27

## Data Availability

Not applicable.
